# Stepping Out of Equilibrium: The Quest for Understanding the Role of Non-Equilibrium (Thermo-)Dynamics in Electronic and Electrochemical Processes

**DOI:** 10.3390/e22091013

**Published:** 2020-09-10

**Authors:** Waldemar Kaiser, Alessio Gagliardi

**Affiliations:** Department of Electrical and Computer Engineering, Technical University of Munich, Karlstraße 45, 80333 Munich, Germany

**Keywords:** non-equilibrium dynamics, non-equilibrium thermodynamics, Kinetic Monte Carlo, molecular electronics, electrochemistry

## Abstract

This editorial aims to interest researchers and inspire novel research on the topic of non-equilibrium Thermodynamics and Monte Carlo for Electronic and Electrochemical Processes. We present a brief outline on recent progress and challenges in the study of non-equilibrium dynamics and thermodynamics using numerical Monte Carlo simulations. The aim of this special issue is to collect recent advances and novel techniques of Monte Carlo methods to study non-equilibrium electronic and electrochemical processes at the nanoscale.

Harvesting energy from renewable sources to satisfy the growing need for energy sustainably is one of society’s key challenges. The design of efficient energy harvesting devices relies on humankind’s knowledge of the underlying physical processes. Since the beginning of the 19th century, the laws of thermodynamics were used to understand the physical behavior and improve the efficiency of energy harvesting devices, mainly steam engines back then. Nowadays, plenty of alternatives, such as solar energy, heat, and synthetic chemical fuels, are used as energy sources. Independent of the energy source, a detailed understanding of the fundamental thermodynamic behavior of electronic and electrochemical processes within energy harvesting devices is required to push the conversion efficiency to the physical limits.

Equilibrium thermodynamics describe a very peculiar state which is still frequently used to describe the fundamental physics of many electronic and electrochemical processes and systems. Interestingly, equilibrium captures their physical behavior with reasonable accuracy. Many systems and processes operate near enough to equilibrium to justify the usage of equilibrium thermodynamic descriptions. However, all systems in nature and technology are driven continuously out of equilibrium by the flux of matter and energy within the system and by the exchange of energy with the environment. This forces the system (more or less far) away from equilibrium. When out of equilibrium, time-dependent information, such as the transport and relaxation of charges or the rates of chemical reactions, becomes relevant. The physics of such non-equilibrium processes and systems determine the performance of most devices ranging from electronic and electrochemical to energy harvesting devices. While equilibrium predictions may allow us to define efficiency limits, non-equilibrium theories can provide insights into the accurate physics of energy conversion.

With the advent of nanotechnologies and nanoscale devices, the accuracy of equilibrium descriptions is to be questioned. Nanoscale devices tend to be surface dominated due to interfaces and nanostructured components. In addition, local inhomogeneities in material properties and rate constants are present in such nanoscale devices. Equilibrium concepts ignore the temporal course of physical processes, and most importantly, neglect local fluctuations in small systems. Non-equilibrium thermodynamic descriptions are required to understand the roles of such fluctuations in time and space, and the roles of the temporal evolution of processes and systems regarding the performances of electronic and electrochemical processes. As the dynamics of processes get even more critical, we need to learn how the temporal evolutions of specific processes affect electronic and electrochemical processes and how they interact with each other. Despite the huge amount of work tackling non-equilibrium thermodynamics in the recent 50 years, a description of non-equilibrium processes remains challenging and requires further effort.

Accessing non-equilibrium properties within experimental measurements is difficult. Resolving the temporal evolution of charge populations requires ultrafast techniques, such as ultrafast transient spectroscopy [1]; the contributions of different species can only be revealed in combination with numerical models. Of even higher complexity is the characterization of physical processes on (sub-)nanometer length scales. Especially in electronic devices, where additional layers hide the information on the electronically active layers, experiments do not allow one to probe the physical properties locally under operation conditions. Computer models of the electronic devices are required to understand the non-equilibrium dynamics and thermodynamics on such small length and time scales effectively.

Kinetic Monte Carlo (kMC) represents a class of powerful numerical models that capture the physics of electronic and electrochemical processes. Starting from fundamental rate equations, kMC allows us to reconstruct the dynamics and thermodynamics of a process or device. First, Monte Carlo methods were developed within the Manhattan project to study neutron diffusion in fissionable materials. One of the best understood Monte Carlo methods is the Metropolis Monte Carlo, which studies equilibrium properties of physical systems [2], while not being capable of including their time evolutions. In contrast, kMC accounts for the time evolution of a physical system. The propagation of the system is modeled via stochastic transitions between microstates i∈S in the multi-dimensional phase space S of the system. The time information of each transition i→j is captured by the transition rates $k_{ji}$. The physics of the studied processes are captured by the transition rates and by the phase space S. kMC essentially mimics the explicit temporal evolution of processes; thus, it is also referred to as “simulated experiments.” On top of that, kMC is a non-equilibrium model by nature. Averaging across trajectories gives us insights into probabilities to occupy certain states. Using such probabilistic information, we can obtain energetic or entropic information. One of the main advantages of kMC with respect to other numerical methods is that it includes local fluctuations in the material properties and transition rates, and the interaction between particles and the environment.

Properties of (nanostructured) materials enter the phase space of the system in the form of sites, e.g., the position of molecules [3] or structure of electronic states [4], or the configuration of reaction sites at nanoparticles [5]. Alternatively, the phase space can be simplified by lattice models, often referred to as lattice-based kMC, to reduce the computational complexity [6,7]. Especially in kMC, not only is the population of particles traced, but also their positions within the physical phase space spanned by the sites are too. The temporal evolution of the system in its phase space of position and energies of all considered particles is captured by the physical transition rates $k_{ji}$. These rates originate from experimental measurements or can be derived starting from underlying quantum mechanical descriptions [8]. Hereby, not only the interactions of particles with the underlying material but also the interactions with other particles and external forces within many-body kMC simulations can be captured. As a result, microscopic properties such as the local charge carrier mobility [3,4] or full device properties such as current-voltage curves [6] or open-circuit voltages in solar cells [9] can be obtained. Thus, kMC acts as a bridge between the atomistic world with device properties on the macroscopic scale [10,11].

Despite the huge amount of work, many crucial physical aspects of electronic and electrochemical devices remain unclear. In organic photovoltaics (OPVs), one of the main fields of our research, the role of non-equilibrium site occupations in the efficiency of charge extraction is studied diversely. Recent studies proposed that non-equilibrium site distributions govern charge transfer at disordered organic heterointerfaces [12]. Giebink et al. showed that the thermalization of charge transfer states under steady-state is incomplete and cannot be described by a Boltzmann distribution [13]. In contrast, Neher et al. observed that equilibrated charge transfer states dominate steady-state recombination [14]. Equilibrium methods frequently describe the thermodynamics of charge separation. The role of entropy [15] and energetic disorder [16] has been demonstrated using equilibrium thermodynamics, while a non-equilibrium picture may be required to fully capture the charge separation process [17]. Charge [18] and exciton transport [19] in organic semiconductors are mainly governed by non-equilibrium dynamics. In electrochemistry, existing kMC studies investigate electrochemical reactions at metal-electrolyte interfaces [5,10], while mass transport is often simplified. Multiscale simulations were recently developed to couple mass transport, simulated by continuum models, with surface reactions obtained by kMC [20]. Still, the effect of the electrolyte in the electrochemical reactions is not well understood.

To cope with these and further challenges, methodological advancements in kMC are required. Mass and electron transport within electrochemical systems occur on very different timescales. Simulating systems with such large discrepancies in timescales requires tremendous computational effort. Acceleration algorithms are required to make simulations of slow processes accessible. Multiscale simulations, which allow insights into different lengths and timescales, are currently under investigation. These are of particular importance to include quantum effects and local material variations in physical transition rates. Recent advances in non-equilibrium thermodynamics descriptions provide interesting methodologies to couple the kinetics of physical processes and local probability distributions. The theory of stochastic thermodynamics especially [21,22], being formulated based on trajectory probabilities, provides a path to study the thermodynamics of microscopic systems subject to local variations.

The special issue “Non-Equilibrium Thermodynamics and Monte Carlo for Electronic and Electrochemical Processes,” aims to cover recent advances and present novel techniques for the usage of Monte Carlo methods in order to study out of equilibrium electronic and electrochemical processes at the nanoscale. In particular, the analyses of the charge and exciton dynamics in molecular electronics, and the reaction dynamics in electrochemical reaction networks, with the help of Monte Carlo methods, are the scope of this Special Issue. Finally, we want to raise questions which are, in our point of view, crucial for the future progress of understanding non-equilibrium dynamics and thermodynamics, especially the role of numerical kMC simulations within these challenges.

1. What are the limits and capabilities of kMC in terms of grasp nanoscale processes and systems?
2. Can we reach a novel level of understanding by merging quantum mechanical and semi-classical dynamics within multiscale descriptions?
3. How can we embrace various orders of magnitude in time/energy and spatial scales?
4. Can kMC combined with non equilibrium thermodynamics provide sufficient insight to design novel materials and devices to overcome the current limitations in performances and efficiencies?
